# Alcohol and Cannabinoids Differentially Affect HIV Infection and Function of Human Monocyte-Derived Dendritic Cells (MDDC)

**DOI:** 10.3389/fmicb.2015.01452

**Published:** 2015-12-22

**Authors:** Marisela Agudelo, Gloria Figueroa, Adriana Yndart, Gianna Casteleiro, Karla Muñoz, Thangavel Samikkannu, Venkata Atluri, Madhavan P. Nair

**Affiliations:** Department of Immunology, Herbert Wertheim College of Medicine, Florida International UniversityMiami, FL, USA

**Keywords:** alcohol, cannabinoids, THC, JWH-015, MDDC, HIV infection

## Abstract

During human immunodeficiency virus (HIV) infection, alcohol has been known to induce inflammation while cannabinoids have been shown to have an anti-inflammatory role. For instance cannabinoids have been shown to reduce susceptibility to HIV-1 infection and attenuate HIV replication in macrophages. Recently, we demonstrated that alcohol induces cannabinoid receptors and regulates cytokine production by monocyte-derived dendritic cells (MDDC). However, the ability of alcohol and cannabinoids to alter MDDC function during HIV infection has not been clearly elucidated yet. In order to study the potential impact of alcohol and cannabinoids on differentiated MDDC infected with HIV, monocytes were cultured for 7 days with GM-CSF and IL-4, differentiated MDDC were infected with HIV-1Ba-L and treated with EtOH (0.1 and 0.2%), THC (5 and 10 μM), or JWH-015 (5 and 10 μM) for 4–7 days. HIV infection of MDDC was confirmed by p24 and Long Terminal Repeats (LTR) estimation. MDDC endocytosis assay and cytokine array profiles were measured to investigate the effects of HIV and substances of abuse on MDDC function. Our results show the HIV + EtOH treated MDDC had the highest levels of p24 production and expression when compared with the HIV positive controls and the cannabinoid treated cells. Although both cannabinoids, THC and JWH-015 had lower levels of p24 production and expression, the HIV + JWH-015 treated MDDC had the lowest levels of p24 when compared to the HIV + THC treated cells. In addition, MDDC endocytic function and cytokine production were also differentially altered after alcohol and cannabinoid treatments. Our results show a differential effect of alcohol and cannabinoids, which may provide insights into the divergent inflammatory role of alcohol and cannabinoids to modulate MDDC function in the context of HIV infection.

## Introduction

Since the discovery of the Human Immunodeficiency Virus (HIV), the acquired immunodeficiency syndrome (AIDS) epidemic has been consistently associated with substance abuse and in some cases substance abuse treatment has been proposed as AIDS prevention (Metzger et al., 1998; Volkow, 2012). Recently, the common problems associated with HIV and substance abuse comorbidities have been extensively discussed as demonstrated by the review of studies in humans and animal models (Chang et al., 2014; Molina et al., 2015). In the context of alcohol abuse and HIV comorbidity, a higher prevalence (42%) of alcohol problems in HIV-infected patients compare to the prevalence in non-HIV patients has been reported using the CAGE questionnaire (Samet et al., 2004). In the context of marijuana abuse and HIV comorbidity, people living with HIV have been reported to have a higher prevalence of cannabis use compared to that of the general population (Braitstein et al., 2001; Furler et al., 2004). For instance, approximately 25% of HIV/AIDS patients have been reported to use marijuana to ameliorate nausea, reduce pain, or as an appetite stimulant (Prentiss et al., 2004); however, the effects of cannabinoids on immune function of these immunocompromised patients remain poorly understood.

Overall, alcohol and drugs of abuse may modulate host–pathogen interactions including immune consequences of HIV infection. To date, it is well known that both the innate and adaptive immune systems are a complex network of cells and cytokines with the primary function of preventing infection. Although the immune system seems to be very resilient, there is evidence of the negative effects of environmental factors such as substances of abuse on immunity (Friedman et al., 2006). Previous studies using animal models have demonstrated a correlation between substance-induced immune dysfunction and greater susceptibility to infections such as in the case of chronic alcohol consumption and susceptibility to SIV infection (Kumar et al., 2005; Molina et al., 2006) or marijuana effects on immune function and ability to decrease host resistance to infections (Klein et al., 2003; Cabral and Staab, 2005; Cabral, 2006). Although substances of abuse have been shown to alter immune functions *in vitro* and in animal models, there is a lack of studies in humans that correlate immunosuppressive effects with increased incidence of infections, including infection with HIV as previously reviewed (Cabral, 2006; Molina et al., 2010a). Therefore, studies on the effects of substances of abuse such as alcohol and marijuana and their immune-modulatory mechanisms of action on HIV infection and disease progression are increasingly on demand since it is evident that the use of recreational substances such as alcohol and marijuana is common in this population and the consequences of substance abuse on HIV infection remain unclear. As previously reviewed most of the literature has highlighted the independent role of alcohol or marijuana on HIV (Chang et al., 2014; Nair and Agudelo, 2014; Molina et al., 2015; Nair et al., 2015); however, a side by side comparison of the *in vitro* effects of alcohol and the role of cannabinoid compounds such as tetrahydrocannabinol (THC) and the synthetic cannabinoid, JWH-015, on HIV infection of monocyte-derived dendritic cells (MDDC) has not been explored.

In the current study, we report, for the first time, the differential effects of substances such as alcohol, THC, and JWH-015, on dendritic cell function after differentiation and following HIV infection *in vitro*. It is hypothesized that alcohol and HIV can exert their effects on MDDC by altering MDDC functions and these effects can be inversely regulated by THC and by the CB_2_ agonist, JWH-015.

## Materials and Methods

### Differentiation of Monocyte-Derived Dendritic Cells (MDDC)

For all the *in vitro* studies, leukopacks were commercially obtained from the community blood bank (One Blood, Miami, FL, USA) from at least three different blood donors. MDDC were prepared from peripheral blood mononuclear cells (PBMC) as previously described by us (Nair et al., 2005, 2009; Agudelo et al., 2013). Briefly, after the separation of adherent and non-adherent cells, monocyte were further purified using the EasySep^TM^ Human Monocyte Enrichment Kit (Stemcell Technologies, catalog #19059), and allowed to differentiate into MDDC by culturing them with complete RPMI media containing 100 U/ml of GM-CSF and 100 U/ml IL-4 (R&D systems, Minneapolis, MN, USA).

### Infection and Treatment of MDDC

**FIGURE 1:**
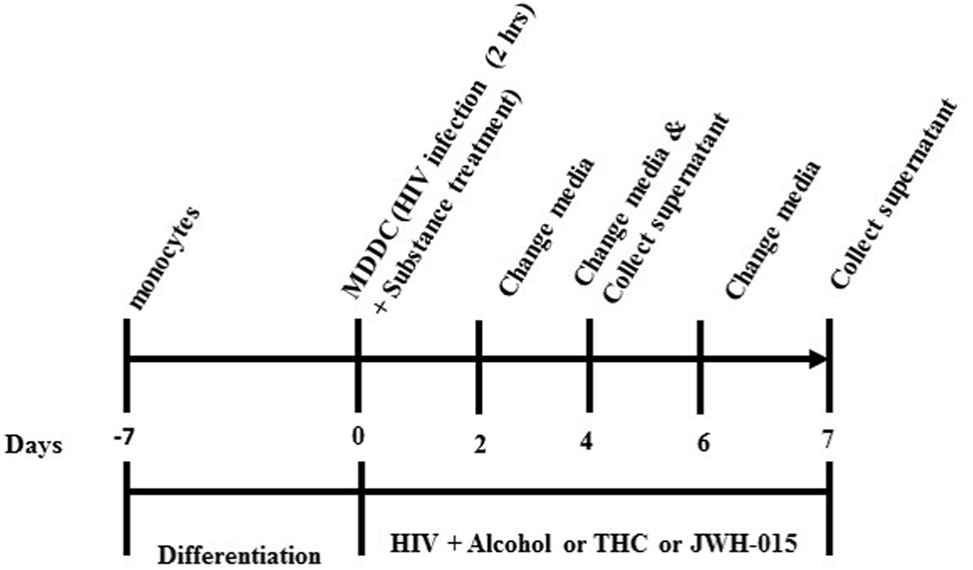
**Experimental design**. Monocytes were allowed to differentiate into MDDC. MDDC were infected with HIV-1 for 2 h followed by treatment with alcohol (0.1 and 0.2%), THC (5 and 10 μM) or JWH-015 (5 and 10 μM). After HIV infection and treatments, MDDC were kept in culture for up to 7 days. Half of media was replenished every 48 h.

After allowing the monocytes to differentiate for 7 days into MDDC using cytokines IL-4 and GM-CSF, the MDDCs were infected and treated. Cells were incubated with polybrene (2 μl/ml) for 30 min prior to infection with HIV-1Ba-L (National Institutes of Health AIDS Research and Reference Reagent Program; catalog no. 510) for 2 h at a concentration of 20 ng/10 million cells. Cells were then washed twice to remove unabsorbed virus and treated with 0.1% (∼20 mM) or 0.2% (∼40 mM) EtOH (Sigma–Aldrich, St. Louis, MO, USA; catalog #E7023), which are equivalent to the physiological blood alcohol concentrations (BAC) of 50 mg/dL, 100 mg/dL, and 200 mg/dL, respectively, and are close to the legal limit for driving under intoxication of 0.08% (80 mg/dL). The cannabinoid group was treated with 5–10 μM of Δ9-THC (Sigma–Aldrich, catalog #T4764) or 5–10 μM of JWH-015 (Tocris Bioscience, Ellisville, MO, USA, catalog #1341), which are concentrations within the range of previous reports using monocytes and macrophages exposed to cannabinoids (Williams et al., 2014; Roth et al., 2015). After HIV infection and treatments, MDDC were kept in culture for up to 7 days. Half of media was replenished every 48 h. Experimental design is provided in **Figure 1**.

### p24 Secretion by ELISA

Concentrations of HIV-1 p24 antigen in HIV-infected MDDC culture supernatants were determined according to the manufacturer’s instructions using commercially available retro-tek HIV-1 p24 antigen ELISA kit (Cat. #0801111; Zeptometrix, Buffalo, NY, USA). ELISA was performed at days 4 and 7 post-infection and post treatment of MDDC to measure secreted p24 levels.

### Intracellular p24 Levels by Flow Cytometry

For intracellular p24 staining, the cells were harvested and counted; equal amounts of cells (1 × 10^6^) were aliquoted in 12 mm × 75 mm polystyrene falcon tubes, catalog #352058 (BD Biosciences, San Jose, CA, USA), blocked with human serum (Chemicon International, Temecula, CA, USA), fixed, and permeabilized with Cytofix/Cytoperm solution (BD Bioscience). The p24 protein was detected with the anti-p24 FITC-labeled antibody (KC57-FITC; Beckman Coulter, Indianapolis, IN, USA). Cells were acquired on an Accuri C6 instrument (BD Accuri, Ann Arbor, MI, USA) and analyzed with FlowJo software (Tree Star, INC, Ashland, OR, USA). A total of 10,000 events were collected for each sample. Cells were gated based on isotype control, anti-IgG1-FITC (Fisher Scientific, Pittsburgh, PA, USA, cat. #1675864). Cells positive for p24 are shown as histogram overlay with shifted mean fluorescence intensity (MFI) compared to controls. Percentages of p24 positive cells and MFI were calculated with FlowJo software.

### HIV Long Terminal Repeats (LTR) Expression by Quantitative Real Time PCR

Expression levels of HIV LTR were analyzed using real-time, quantitative PCR. Reverse transcription was performed with the 2720 Thermal cycler (Applied Biosystems) using aliquots of total RNA extracted from samples. The cDNA samples were diluted to 20 ng/ul and gene-specific primers were used: forward and reverse LTR HIV-1 primers and Taqman LTR-HIV (Cat. #VC00021 and VC00023, Sigma–Aldrich). To normalize the fold changes in gene expression, two internal control genes were used, GAPDH Taqman primer (Cat. #4331182, Life technologies) and 18S rRNA (Cat. #4333760F, Applied Biosystems). Fold changes were calculated based on uninfected control. All real-time PCR reactions were performed using the MX3005P detection system (Agilent Technologies) and the amplifications were done using the Brilliant II QPCR Master Mix (Cat. #600804, Agilent Technologies). The thermal cycling conditions were composed of an initial denaturation step at 95°C for 10 min, 45 cycles at 95°C for 30 s and 55°C for 1 min. The experiments were carried out in duplicates for each data point. The relative quantification in gene expression was determined using the 2^-ΔΔ^*^C^*^t^ method (Livak and Schmittgen, 2001).

### Endocytosis Assay

After MDDC differentiation, cells were treated with alcohol, THC or JWH-015 for 24 h. Then, the cells were cultured with FITC-dextran (1 mg/ml) in PBS + 1% FBS for 1 h at 37°C. The assay was terminated by adding ice-cold PBS. Cell pellets were washed three times with PBS and the MDDC immediately analyzed for accumulation of intracellular fluorescence in a Biotek plate reader (Winooski, VT, USA) at 490 nm excitation and 520 nm emission. In addition, MDDC infected with HIV and treated with alcohol, THC, or JWH-015, were also analyzed to assess their endocytic capacity.

### Cytokine Arrays

Supernatants were collected at 7 days post-infection from MDDC infected with HIV and treated with either EtOH, THC, or JWH015. The expression of 48 inflammatory cytokines was analyzed as per manufacture recommendations with the RayBiotech inflammation arrays (catalog #AAH-INF-3-8, RayBiotech, Norcross, GA, USA). Data are expressed as fold change of cytokines secreted by HIV-infected and treated MDDC compared to the cytokines secreted by HIV-infected MDDC. Chemiluminescence signals were detected by a film developer and analyzed by densitometry using Image J software. Data were further analyzed using the RAYBIO Analysis Tool.

### Statistical Analysis

Results are presented as mean ± SEM, and *p*-values <0.05 are considered significant. Data were analyzed using Prism software (GraphPad Software, La Jolla, CA, USA), and statistical significance was determined by performing unpaired two-tailed Student’s *t*-test or ANOVA followed by the Dunnett’s post-test. All *in vitro* studies were performed with cells from at least three different donors with multiple replicates (at least two).

## Results

### Alcohol and Cannabinoids Differentially Modulate p24 Production by MDDC

**FIGURE 2:**
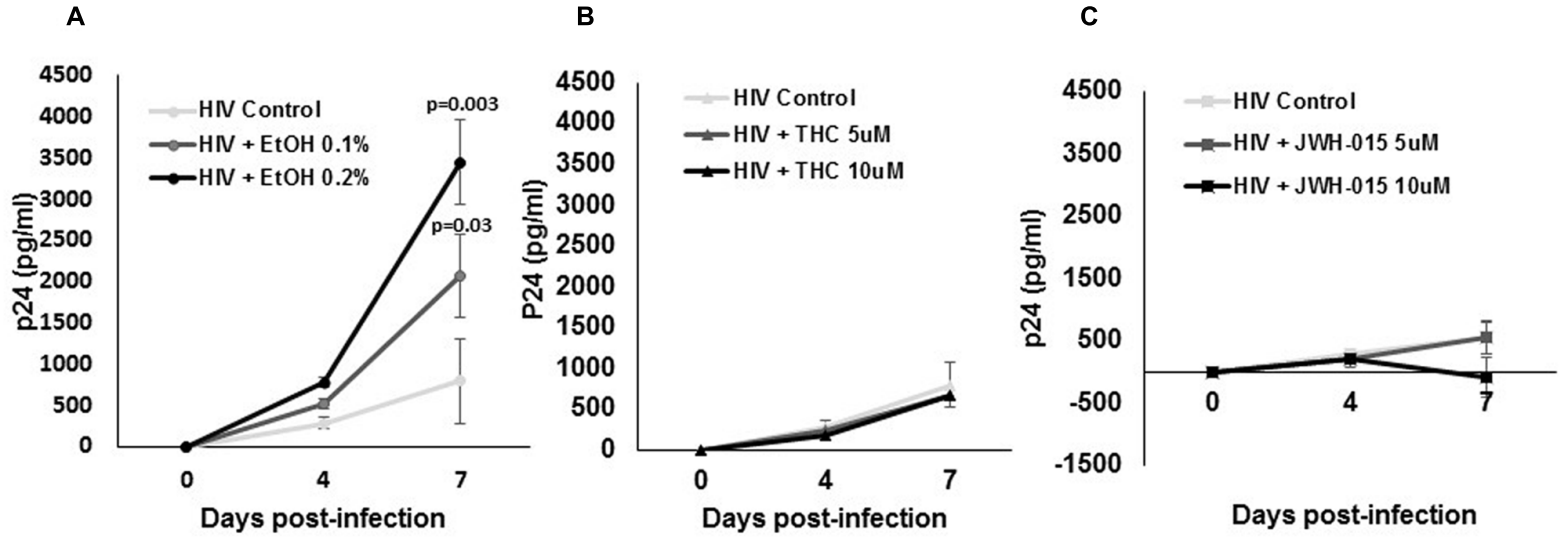
**Alcohol and cannabinoids differentially modulate p24 production by MDDC**. MDDC were infected with HIV and treated with **(A)** alcohol, **(B)** THC, or **(C)** JWH-015 for up to 7 days. Supernatants were collected at days 4 and 7 post-infection and analyzed by ELISA to measure p24 levels. Data are expressed as pg/ml of p24 levels of five individual experiments performed at least in duplicates. Significant *p*-values are noted on graphs, *p* ≤ 0.05 is considered significant.

To determine if alcohol, THC, and JWH-015 modulate HIV-1 replication, MDDC were infected by the macrophage tropic replication competent strain, HIV-1Ba-L, followed by treatment with alcohol, THC, or JWH-015 using the experimental design (described in Materials and Methods, **Figure 1**). Although our ELISA results have demonstrated an increase in p24 levels over time after HIV infection (0–7 days), only the alcohol treated cells showed a significant dose dependent increase in p24 levels at 7 days post-infection (**Figure 2A**) while both concentrations (5 and 10 μM) of THC treatment had no major effect on p24 levels when compared to the HIV infected control (**Figure 2B**). Surprisingly, treatment with 10 μM of the synthetic cannabinoid, JWH-015, caused a decrease in p24 levels by 7 days (**Figure 2C**).

### Alcohol and Cannabinoids Differentially Modulate Intracellular Levels of p24 and the Percentage of HIV Positive MDDC

**FIGURE 3:**
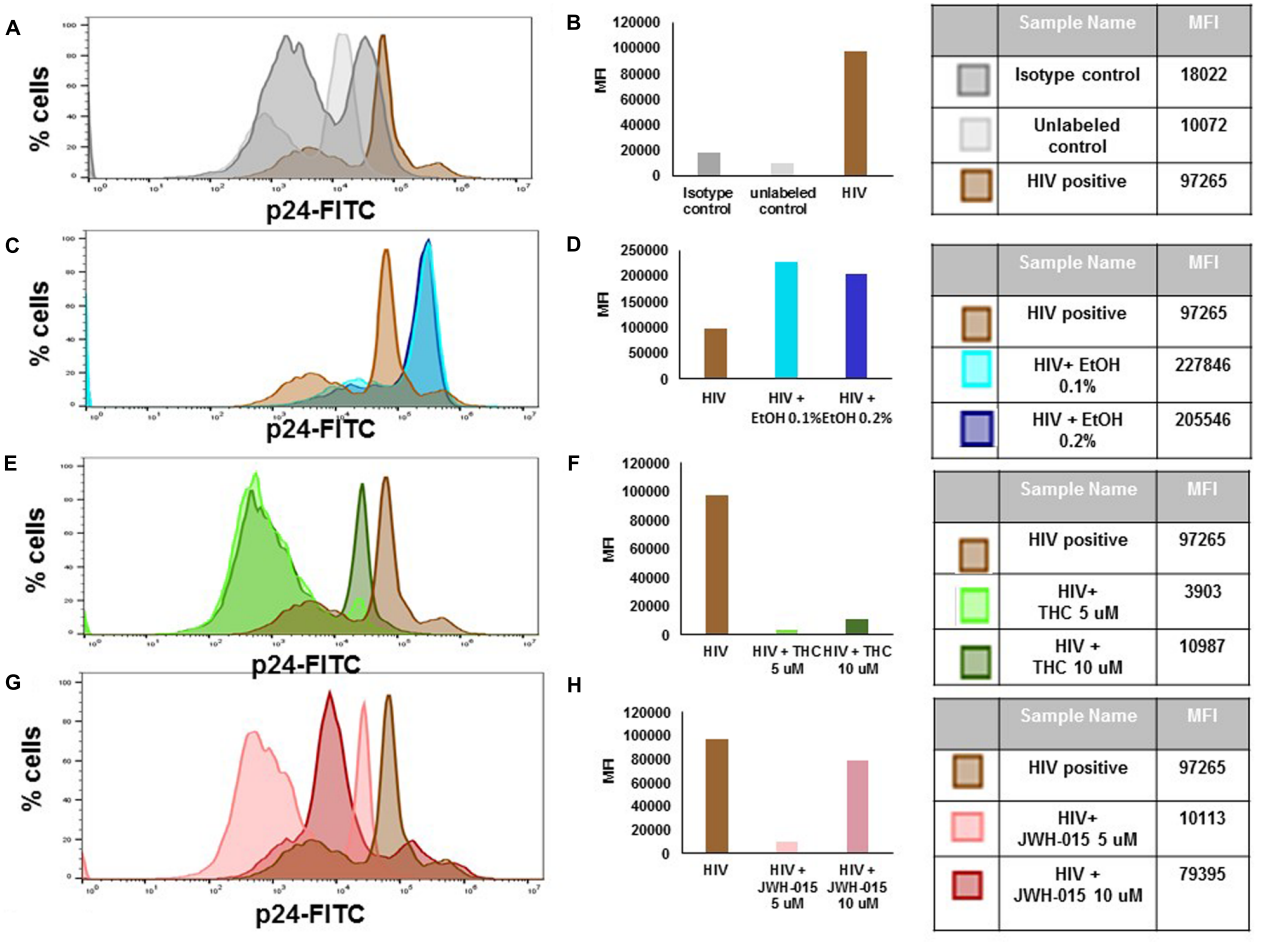
**Alcohol and cannabinoids differentially modulate intracellular levels of p24 in MDDC**. MDDC were infected with HIV and treated with alcohol, THC or JWH-015 for up to 7 days prior to assessing intracellular p24 levels by flow cytometry. A representative histogram overlay is shown in this figure **(A,C,E,G)**. Mean Fluorescence Intensity (MFI) corresponding to histogram overlays were calculated using FlowJo software and are shown in this figure **(B,D,F,H)**. A total of 10,000 events were collected for each sample. Cells were gated based on isotype control. Data are expressed as % of p24 positive cells and MFI of FITC-labeled p24 and are representative of three individual experiments performed at least in duplicates.

**FIGURE 4:**
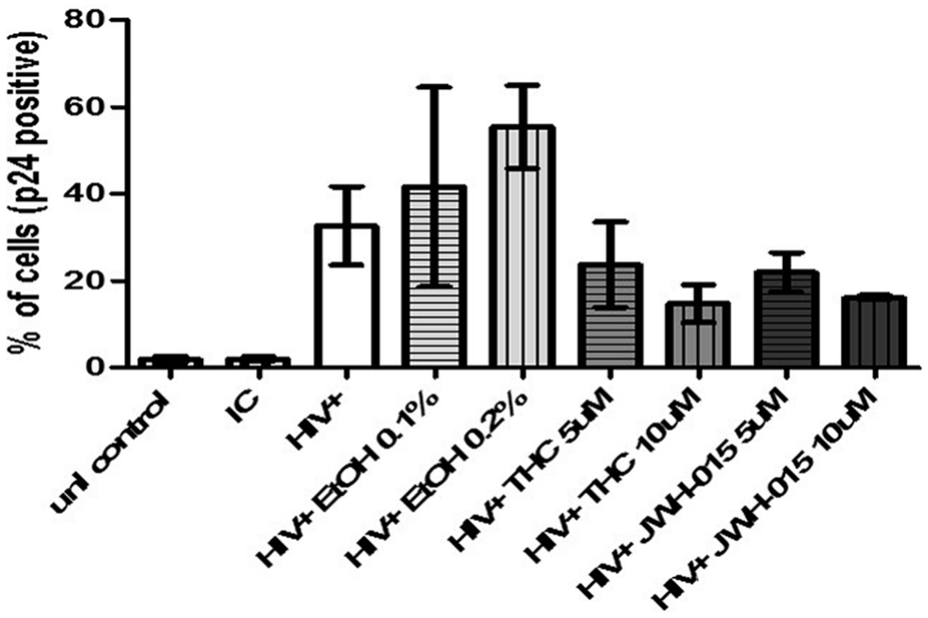
**Alcohol and cannabinoids differentially modulate the percentage of HIV positive MDDC**. MDDC were infected with HIV and treated with alcohol, THC or JWH-015 for up to 7 days prior to assessing intracellular p24 levels by flow cytometry. Bar graphs correspond to the percentage of MDDC positive for p24 antigen. A total of 10,000 events were collected for each sample. Cells were gated based on isotype control. Data are expressed as % of p24 positive cells of three individual experiments.

Flow cytometry analysis of p24 protein levels revealed an increase in both mean fluorescent intensity (MFI; **Figures 3A,B**) and in the percentage of cells expressing intracellular levels of the viral p24 core antigen (**Figure 4**) after 7 days post-infection confirming the internalization of virus and production of p24 core antigen by MDDC. In addition, both concentrations of alcohol, 0.1 and 0.2%, (**Figures 3C,D**) induced more than twofold increase in MFI when compared to HIV control, while the cannabinoids caused a reduction in MFI (**Figures 3E–H**) when compared to HIV control. Although there were visible differences in the modulation of MFI (**Figures 3B,D,F,H**) and in the percentage of cells expressing intracellular p24 levels (**Figure 4**) by alcohol and cannabinoids (THC and JWH-015), the effects observed were not statistically significant.

### HIV-Infection Rate of MDDC is Increased in the Presence of Alcohol and THC

**FIGURE 5:**
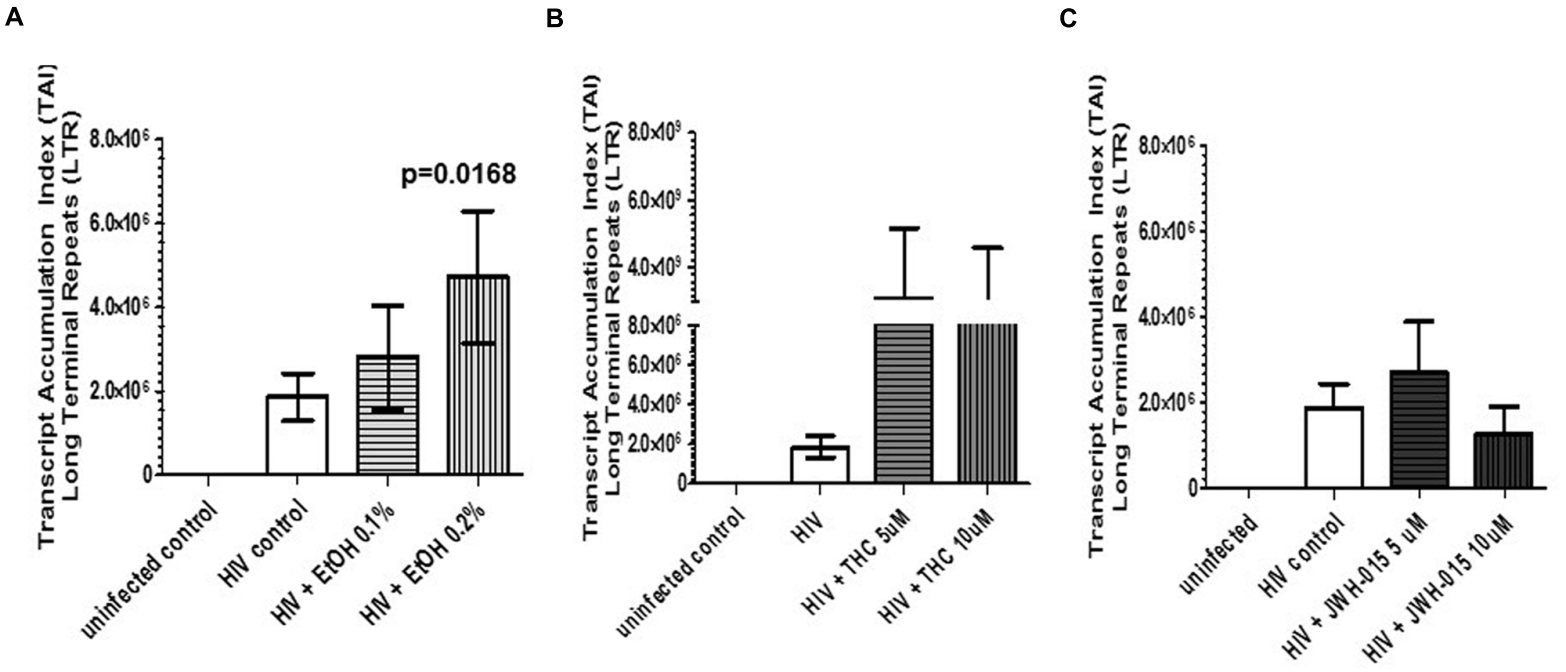
**HIV-infection rate of MDDC is increased in the presence of alcohol and THC**. MDDC were infected with HIV and treated with **(A)** alcohol, **(B)** THC, or **(C)** JWH-015 for up to 7 days prior to assessment of HIV LTR by real-time PCR. Data are expressed as Transcript Accumulation index (TAI). To normalize the fold changes in gene expression, two internal control genes were used, GAPDH and 18S rRNA. Fold changes were calculated based on uninfected control. Data are representative of three individual experiments performed at least in duplicates.

Assessment of HIV LTR by real-time PCR, confirmed HIV integration and infectivity of MDDC. Overall, we observed a dose dependent increase in HIV LTR in alcohol-treated HIV-infected MDDC compared to the untreated HIV-infected cells; however, only the higher concentration of alcohol (0.2%) induced a significant expression of LTR (**Figure 5A**). Although THC also caused an induction in LTR when compared to control and alcohol treated cells, it was not statistically significant (**Figure 5B**). The synthetic cannabinoid, JWH-015 did not affect LTR expression when compared to HIV control, in contrast, there was a decrease in LTR after treatment with 10 μM of JWH-015 (**Figure 5C**).

### Alcohol Treatment Induces Higher Levels of Endocytosis by Uninfected MDDC When Compare to Control and Cannabinoid Treated Cells

**FIGURE 6:**
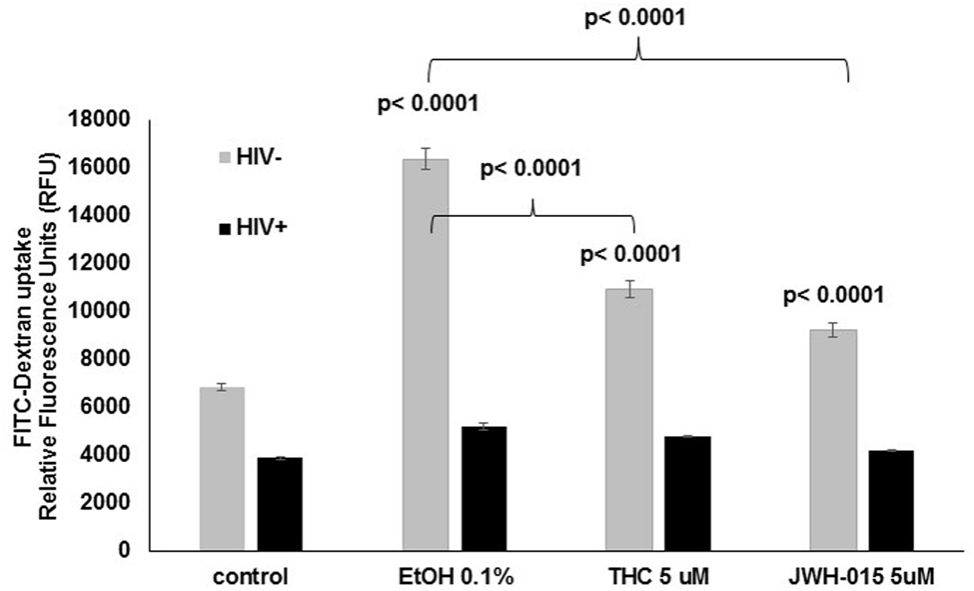
**Alcohol treatment induces higher levels of endocytosis by uninfected MDDC when compare to control and cannabinoid treated cells**. After MDDC differentiation, cells were treated with alcohol, THC or JWH-015 for 24 h. In addition, MDDC infected with HIV followed by treatment with alcohol, THC, or JWH-015 were also analyzed for FITC-Dextran uptake. Data are expressed as relative fluorescence units (RFU) representing FITC-Dextran uptake by uninfected (light gray bars) and infected (black bars) MDDC. Data are representative of three individual experiments performed at least in duplicates. Significant *p*-values are noted on graphs, *p* ≤ 0.05 is considered significant.

Receptor mediated endocytosis was measured prior to HIV infection and after HIV infection by the uptake of FITC-dextran. Our results show a significant increase of FITC-dextran intake by MDDC that had been treated with alcohol (0.1%), THC (5 μM), or JWH-015 (5 μM; **Figure 6**). Although all treatments increased endocytosis in uninfected cells, THC and JWH-015 treated cells showed significantly lower levels of endocytosis when compared to alcohol treated MDDC. The overall increase in endocytosis by the substance abuse treated cells was only observed prior to HIV infection since after the cells were infected with HIV, there was an overall decrease in endocytosis with no differences across the groups.

### Alcohol and Cannabinoids Differentially Modulate MDDC Cytokine Profiles During HIV Infection

**FIGURE 7:**
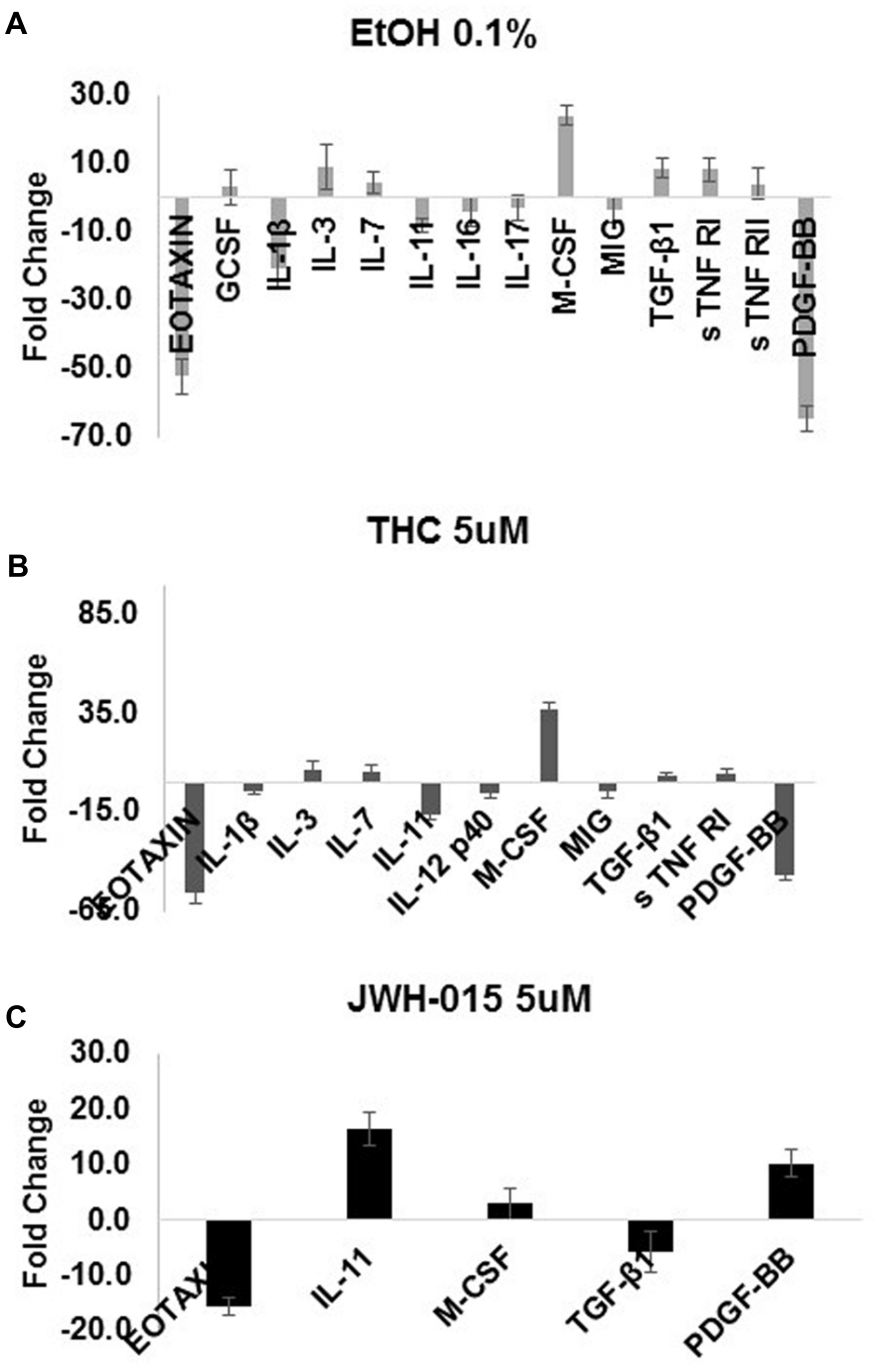
**Alcohol and cannabinoids differentially modulate MDDC cytokine profiles during HIV infection**. The production of cytokines by MDDC was assessed after HIV infection and stimulation with alcohol (0.1%), THC (5 μM) or JWH-015 (5 μM). Supernatants were collected at 7 days post-infection and used to investigate the expression of 48 inflammatory cytokines with the RayBiotech inflammation arrays. A summary of the cytokines that were modulated (≥ threefold) during HIV infection and substance abuse treatment are shown in this figure **(A)** 0.1% alcohol, **(B)** 5 μM THC, and **(C)** 5 μM JWH-015. Data are expressed as fold change of cytokines secreted by HIV-infected and treated MDDC compared to the cytokines secreted by HIV-infected MDDC. Chemiluminescence signals were analyzed by densitometry using Image J software. Data were further analyzed using the RAYBIO Analysis Tool.

The production of cytokines was assessed after HIV infection and stimulation with alcohol, THC or JWH-015. A summary of the cytokines that were modulated (≥ threefold) during HIV infection and substance abuse treatment are shown in **Figure 7** (A) 0.1% alcohol, (B) 5 μM THC, and (C) 5 μM JWH-015. Treatment of HIV-infected MDDC with 0.1% alcohol caused an induction of seven cytokines (G-CSF, IL-3, IL-7, M-CSF, TGF-β1, s TNF RI, and s TNF RII), while treatment with 5 μM THC induced the levels of five cytokines (IL-3, IL-7, M-CSF, TGF-β1, s TNF RI), and treatment with 5 μM JWH-015 upregulated only three cytokines (IL-11, M-CSF, and PDGF-BB). Cytokine downregulation was also observed across the groups, 0.1% alcohol treatment downregulated seven cytokines (Eotaxin, IL-1β, IL-11, IL-16, IL-17, MIG, and PDGF-BB), while 5 μM THC treatment downregulated six cytokines (Eotaxin, IL-1β, IL-11, IL-12 p40, MIG, PDGF-BB), and treatment with 5 μM JWH-015 caused downregulation of two cytokines (Eotaxin and TGF-β1). Specific fold differences between treatments and HIV control are highlighted in **Table 1**.

**Table 1:** Summary of the effects of alcohol and cannabinoids, THC and JWH-015 on the modulation of cytokine production by HIV-infected MDDC.

Cytokine	EtOH 0.1%	THC 5 μ M	JWH-015 5 μ M
EOTAXIN	–52.3	–55.2	–15.5
GCSF	3.0	2.8	0.6
ICAM-1	1.5	1.6	1.1
I-309	0.6	1.8	2.0
IL-1β	–20.5	–4.0	2.1
IL-3	9.1	6.9	2.3
IL-6sR	2.5	2.6	1.6
IL-7	4.4	5.6	0.1
IL-11	–8.3	–16.2	16.7
IL-12 p40	–1.4	–5.5	1.4
IL-12 p70	0.3	0.0	2.4
IL-16	–4.1	–1.4	1.1
IL-17	–3.1	–1.5	1.5
M-CSF	24.1	37.1	3.1
MIG	–3.6	–4.2	1.7
MIP-1α	2.7	2.9	2.0
MIP-1δ	1.1	3.0	1.7
TGF-β1	8.7	3.3	–5.6
TNF-α	–0.9	0.5	2.1
TNF-β	–2.6	–0.4	2.7
s TNF RI	8.3	4.9	–0.7
s TNF RII	4.1	2.5	0.1
PDGF-BB	–64.9	–46.4	10.4

*Cytokine array was conducted using supernatants from HIV-infected MDDC treated with alcohol (0.1%), THC (5 μM), of JWH-015 (5 μM). A summary of cytokines with more than threefold increase (light gray) or decrease (dark gray) are highlighted in the table. Data are expressed as fold change of cytokines secreted by HIV-infected and treated MDDC compared to the cytokines secreted by HIV-infected MDDC*.

## Discussion

Our study supports evidence of a major role of MDDC in the enhancement of HIV infection and integration *in vitro*. Although the MDDC role during HIV infection has been somehow controversial due to the discrepancy regarding HIV infection of MDDC and evidence showing that HIV spreads from virus-containing MDDC (not necessarily productively infected) to T cells via an infectious synapse (McDonald et al., 2003; Blanchet et al., 2011), MDDC are professional antigen presenting cells and they do get productively infected with HIV as shown by previous reports (Scott-Algara et al., 2008) and our own current findings demonstrating an increase in p24 intracellular levels, p24 secretion, and LTR. Despite MDDC questionable ability to get productively infected with HIV, they play a major role during HIV-1 *trans* infection of CD4^+^ T cells as previously reviewed (Agudelo et al., 2010; Rinaldo, 2013). Moreover, there is evidence of the ability of circulating DC to purge HIV-1 from latency and re-initiate virus replication in proliferating primary T lymphocytes (van der Sluis et al., 2013).

To the best of our knowledge, this is the first report that performs a side by side comparison of the *in vitro* effects of alcohol and cannabinoids in MDDC infected with HIV. We analyzed the effects of alcohol, THC, and JWH-015 on HIV infection and integration by measuring p24 production, intracellular p24 levels, and LTR. In addition, the immune-modulatory effects of these substances on MDDC function under HIV infection were also evaluated as measured by endocytosis and cytokine production. Our results show *in vitro* alcohol treatment of MDDC increased p24 secretion (**Figure 2A**), intracellular p24 levels (**Figures 3C,D** and **4**), percentage of cells expressing p24, and LTR (**Figure 5A**). Our findings are in accordance with previous reports showing alcohol enhances HIV-1 infection of human monocyte-derived cells; however, those reports were performed in cord blood monocyte-derived macrophages (Mastrogiannis et al., 2014) and in blood monocyte-derived macrophages (Wang et al., 2002). Furthermore, alcohol ingestion has been shown to increase HIV-1 replication in human PBMC, which may be due to alcohol-induced functional impairment of various subsets of lymphocytes in the peripheral blood (Bagasra et al., 1993). In addition, substantial evidence from animal (Kumar et al., 2005; Molina et al., 2006; Sarkar and Chang, 2013) and human studies (Míguez-Burbano et al., 2009; Baum et al., 2010; Molina et al., 2014) has demonstrated that alcohol has an immune-modulatory effect during HIV disease progression and has the ability to disrupt HAART (Míguez-Burbano et al., 2009). More specific associations between alcohol consumption and lower levels of CD4 T cells have been reported among HIV-infected alcoholic patients (Samet et al., 2007; Baum et al., 2010). However, additional studies are necessary to elucidate the mechanisms of alcohol-induced immune-modulation in the context of HIV infection.

In contrast to alcohol results, when the HIV-infected MDDC were treated with the main psychoactive component of marijuana, THC, there were no changes in p24 secretion (**Figure 2B**). However, there was an evident decrease in p24 intracellular levels (**Figures 3E,F**) and in the percentage of cells expressing p24 (**Figure 4**) implying a reduction in HIV infection. Surprisingly, when LTR levels were examined, integration seemed to be elevated since THC acted similar to alcohol by inducing LTR (**Figure 5B**) levels; although, the induction had high variability between samples and was not significant. In contrast, a previous report using THC immediately prior to or continuously following HIV-1 exposure failed to alter infection (Williams et al., 2014). However, their study was performed with primary human monocytes and treatment was performed during differentiation of monocytes. Although in different cells and under different treatment conditions, some of their findings (Williams et al., 2014) are somehow consistent to our results showing that THC reduced the number of p24 positive cells (**Figure 4**) with little to no effect on virus production (**Figure 2B**). It is relevant to point out that in our *in vitro* findings, there were no differences in p24 secretion when we compared THC treated HIV-infected MDDC with non-treated HIV-infected MDDC. Similarly, animal studies have shown that simian immunodeficiency virus (SIV)-infected female rhesus macaques exposed to chronic administration of THC prior to and following SIV infection exhibited no changes in markers of SIV disease, including viral load (Amedee et al., 2014). However, there were contrasting effects of chronic THC exposure in males versus females, with males having lower plasma and CSF viral loads (Molina et al., 2010b).

In an effort to elucidate the mechanism of THC suppression of HIV-1 infection, we also performed treatment with CB_2_ agonist, JWH-015. HIV-infected MDDC treated with JWH-015 showed a decrease in p24 secretion by 7 days post-infection (**Figure 2C**), a decrease in p24 intracellular levels (**Figures 3G,H**) and a decrease in the percentage of p24 positive cells (**Figure 4**) when compared with the HIV-infected control or the HIV-infected alcohol treated cells. We also observed a decrease in LTR levels after treatment with 10 μM JWH-015 when compared with HIV-infected control and HIV-infected alcohol or cannabinoid treated cells. These findings may support a major role of CB_2_ receptors in the HIV replication machinery, and these results are in accordance with previous findings with macrophages revealing a marked decrease in HIV-1 LTR activation by the CB_2_ ligands, JWH133, GP1a, O-1966 and showing a significant decrease in RT activity when a CB_2_ agonist was present (Ramirez et al., 2013). Taken together, these results using MDDC and previous findings with macrophages (Ramirez et al., 2013) indicate that CB_2_ may limit HIV-1 infection in human monocyte-derived cells. Furthermore, a similar study with JWH-133, ACEA, and O-1602 indicated that the ability of THC to reduce macrophage susceptibility to HIV infection during monocyte differentiation was mediated primarily through CB_2_ (Williams et al., 2014).

Besides modulation of HIV infection of MDDC, our results demonstrate the ability of alcohol and cannabinoids (THC and JWH-015) to affect DC function as shown by an increase in endocytosis in uninfected cells and an overall decrease in the endocytic capacity after HIV infection (**Figure 6**). These findings may be explained by the capacity of immature dendritic cells to be highly endocytic (Garrett et al., 2000) and the ability of HIV-1_BaL_ to induce partial maturation of dendritic cells (Mercier et al., 2013). Therefore, based on our results and previous literature (Mercier et al., 2013) the observed decrease in endocytosis after HIV infection and the attenuation of the substance abuse-induction of endocytosis might be due to the maturation status of the MDDC. We are the first ones to show an induction of endocytosis by healthy uninfected MDDC treated with alcohol or cannabinoids. Previous literature in the context of alcohol has demonstrated the ability of alcohol to modulate endocytosis; however, the reports are in rat hepatocytes, in which ethanol impaired receptor-mediated endocytosis (Casey et al., 1989; Tuma et al., 1991). In the context of cannabinoids, there is evidence demonstrating that exposure to THC impairs the capacity for receptor-mediated endocytosis by human dendritic cells (Roth et al., 2015); however, their immunoregulatory effects on human MDDC were observed at lower THC concentrations (0.5–3.2 μM). Other findings in mice, have demonstrated that under certain conditions, THC enhances HIV antigen-specific immune responses (Chen et al., 2015).

In the past, alcohol and cannabinoids have been shown to alter several aspects of immune function including cytokine production and lymphocyte phenotype (Friedman et al., 1995, 2003; Szabo, 1999; Klein et al., 2003; Szabo et al., 2004; Crews et al., 2006; Molina et al., 2010a). According to the cytokine analysis performed (**Figure 7** and **Table 1**), alcohol and cannabinoids induced differential cytokine production by HIV-infected MDDC and caused a major immune-modulatory effect, which may exacerbate HIV infection and pathogenesis. When compared to HIV control, alcohol (0.1%) and THC (5 μM) treatments had similar effects and induced IL-3, IL-7, M-CSF, TGF-β1, sTNF RI, and sTNF RII while decreasing IL-1β, IL-11, IL-12p40, TNFβ, and PDGF-BB. However, the JWH-015 treatments had opposing effects as shown by an increase on IL-1β, IL-11, IL-12p40, and IL-12 p70, MCSF, MIP1α, TNFα, TNFβ, and PDGF-BB while reducing only TGF-β1 and sTNF RI. All three substances, alcohol (–52.3), THC (–55.2) and JWH-015 (–15.5), remarkably reduced the levels of eotaxin, which is a chemokine associated with lower susceptibility to infection in macaques (Promadej-Lanier et al., 2010). Another unique difference observed was on the effects on IL-11 production after alcohol (–8.3), THC (–16.2), and JWH-015 (16.7) treatments. This is relevant since IL-11 may reduce HIV-1-mediated immune activation while achieving elimination of virally infected cells as previously reported (Favors et al., 2012). Lastly, the effects on PDGF-BB were also different between alcohol (–64.9), THC (–46.4) versus JWH-015 (10.4) treatments. This is relevant within the HIV field since platelet-derived growth factor (PDGF) is one of the toxic mediators shown to be upregulated in the brains of macaques with SIV encephalitis and shown to play a major role in HIV neuro-pathogenesis (Potula et al., 2004; Bethel-Brown et al., 2011).

Overall, the differential immune-modulatory effects of alcohol and cannabinoids could be explained by the different mechanisms of action of these substances since the effects of marijuana on the immune system have been demonstrated to be receptor-mediated, occurring directly via specific receptors on immune cells (Friedman et al., 2003). For instance, THC exerts its effects through both CB_1_ and CB_2_ receptors (Matsuda et al., 1990; Munro et al., 1993), while JWH-015 acts as a CB_2_ agonist (Huffman, 2000). Unlike cannabinoids and other addictive drugs of abuse, alcohol, does not appear to bind to a specific receptor (Peter et al., 2008). In summary alcohol and cannabinoids have been shown to induce neuro-immune-modulatory consequences during healthy conditions and during HIV infection and disease progression as highlighted in several recent reviews (Chang et al., 2014; Molina et al., 2015; Nair et al., 2015). Our current findings were able to highlight a divergent role of both substances in HIV infection and integration of human MDDC, and along with future studies, will allow us to elucidate the immunological consequences of alcohol and/or cannabinoids on the innate and adaptive immune system of an immune-compromised host. However, additional studies are still necessary to elucidate the molecular mechanisms involving alcohol and/or cannabinoid induced effects in the context of HIV infection.

## Author Contributions

MA contributed to the experimental design of the project, perform experiments, perform data analysis, and wrote the manuscript, GF and GC performed experiments, AY and KM provided technical assistance and advice with experimental troubleshooting, TS provided advice and guidance regarding HIV experiments, VA provided assistance with HIV experiments, LTR estimation and data analysis, MN provided financial support and guidance with HIV experimental design.

## Conflict of Interest Statement

The authors declare that the research was conducted in the absence of any commercial or financial relationships that could be construed as a potential conflict of interest.
